# Transgender Women in the Female Category of Sport: Perspectives on Testosterone Suppression and Performance Advantage

**DOI:** 10.1007/s40279-020-01389-3

**Published:** 2020-12-08

**Authors:** Emma N. Hilton, Tommy R. Lundberg

**Affiliations:** 1 Faculty of Biology, Medicine and Health, University of Manchester, Manchester, UK; 2 Department of Laboratory Medicine/ANA Futura, Division of Clinical Physiology, Karolinska Institutet, Alfred Nobles Allé 8B, Huddinge, 141 52 Stockholm, Sweden; 3 Unit of Clinical Physiology, Karolinska University Hospital, Stockholm, Sweden

## Abstract

Males enjoy physical performance advantages over females within competitive sport. The sex-based segregation into male and female sporting categories does not account for transgender persons who experience incongruence between their biological sex and their experienced gender identity. Accordingly, the International Olympic Committee (IOC) determined criteria by which a transgender woman may be eligible to compete in the female category, requiring total serum testosterone levels to be suppressed below 10 nmol/L for at least 12 months prior to and during competition. Whether this regulation removes the male performance advantage has not been scrutinized. Here, we review how differences in biological characteristics between biological males and females affect sporting performance and assess whether evidence exists to support the assumption that testosterone suppression in transgender women removes the male performance advantage and thus delivers fair and safe competition. We report that the performance gap between males and females becomes significant at puberty and often amounts to 10–50% depending on sport. The performance gap is more pronounced in sporting activities relying on muscle mass and explosive strength, particularly in the upper body. Longitudinal studies examining the effects of testosterone suppression on muscle mass and strength in transgender women consistently show very modest changes, where the loss of lean body mass, muscle area and strength typically amounts to approximately 5% after 12 months of treatment. Thus, the muscular advantage enjoyed by transgender women is only minimally reduced when testosterone is suppressed. Sports organizations should consider this evidence when reassessing current policies regarding participation of transgender women in the female category of sport.

**Supplementary Information:**

The online version contains supplementary material available at 10.1007/s40279-020-01389-3.

## Key Points

**Table:** 

Given that biological males experience a substantial performance advantage over females in most sports, there is currently a debate whether inclusion of transgender women in the female category of sports would compromise the objective of fair and safe competition.
Here, we report that current evidence shows the biological advantage, most notably in terms of muscle mass and strength, conferred by male puberty and thus enjoyed by most transgender women is only minimally reduced when testosterone is suppressed as per current sporting guidelines for transgender athletes.
This evidence is relevant for policies regarding participation of transgender women in the female category of sport.

## Introduction

Sporting performance is strongly influenced by a range of physiological factors, including muscle force and power-producing capacity, anthropometric characteristics, cardiorespiratory capacity and metabolic factors [1, 2]. Many of these physiological factors differ significantly between biological males and females as a result of genetic differences and androgen-directed development of secondary sex characteristics [3, 4]. This confers large sporting performance advantages on biological males over females [5].

When comparing athletes who compete directly against one another, such as elite or comparable levels of school-aged athletes, the physiological advantages conferred by biological sex appear, on assessment of performance data, insurmountable. Further, in sports where contact, collision or combat are important for gameplay, widely different physiological attributes may create safety and athlete welfare concerns, necessitating not only segregation of sport into male and female categories, but also, for example, into weight and age classes. Thus, to ensure that both men and women can enjoy sport in terms of fairness, safety and inclusivity, most sports are divided, in the first instance, into male and female categories.

Segregating sports by biological sex does not account for transgender persons who experience incongruence between their biological sex and their experienced gender identity, and whose legal sex may be different to that recorded at birth [6, 7]. More specifically, transgender women (observed at birth as biologically male but identifying as women) may, before or after cross-hormone treatment, wish to compete in the female category. This has raised concerns about fairness and safety within female competition, and the issue of how to fairly and safely accommodate transgender persons in sport has been subject to much discussion [6–13].

The current International Olympic Committee (IOC) policy [14] on transgender athletes states that “it is necessary to ensure insofar as possible that trans athletes are not excluded from the opportunity to participate in sporting competition”. Yet the policy also states that “the overriding sporting objective is and remains the guarantee of fair competition”. As these goals may be seen as conflicting if male performance advantages are carried through to competition in the female category, the IOC concludes that “restrictions on participation are appropriate to the extent that they are necessary and proportionate to the achievement of that objective”.

Accordingly, the IOC determined criteria by which transgender women may be eligible to compete in the female category. These include a solemn declaration that her gender identity is female and the maintenance of total serum testosterone levels below 10 nmol/L for at least 12 months prior to competing and during competition [14]. Whilst the scientific basis for this testosterone threshold was not openly communicated by the IOC, it is surmised that the IOC believed this testosterone criterion sufficient to reduce the sporting advantages of biological males over females and deliver fair and safe competition within the female category.

Several studies have examined the effects of testosterone suppression on the changing biology, physiology and performance markers of transgender women. In this review, we aim to assess whether evidence exists to support the assumption that testosterone suppression in transgender women removes these advantages. To achieve this aim, we first review the differences in biological characteristics between biological males and females, and examine how those differences affect sporting performance. We then evaluate the studies that have measured elements of performance and physical capacity following testosterone suppression in untrained transgender women, and discuss the relevance of these findings to the supposition of fairness and safety (i.e. removal of the male performance advantage) as per current sporting guidelines.

## The Biological Basis for Sporting Performance Advantages in Males

The physical divergence between males and females begins during early embryogenesis, when bipotential gonads are triggered to differentiate into testes or ovaries, the tissues that will produce sperm in males and ova in females, respectively [15]. Gonad differentiation into testes or ovaries determines, via the specific hormone milieu each generates, downstream in utero reproductive anatomy development [16], producing male or female body plans. We note that in rare instances, differences in sex development (DSDs) occur and the typical progression of male or female development is disrupted [17]. The categorisation of such athletes is beyond the scope of this review, and the impact of individual DSDs on sporting performance must be considered on their own merits.

In early childhood, prior to puberty, sporting participation prioritises team play and the development of fundamental motor and social skills, and is sometimes mixed sex. Athletic performance differences between males and females prior to puberty are often considered inconsequential or relatively small [18]. Nonetheless, pre-puberty performance differences are not unequivocally negligible, and could be mediated, to some extent, by genetic factors and/or activation of the hypothalamic–pituitary–gonadal axis during the neonatal period, sometimes referred to as “minipuberty”. For example, some 6500 genes are differentially expressed between males and females [19] with an estimated 3000 sex-specific differences in skeletal muscle likely to influence composition and function beyond the effects of androgenisation [3], while increased testosterone during minipuberty in males aged 1–6 months may be correlated with higher growth velocity and an “imprinting effect” on BMI and bodyweight [20, 21]. An extensive review of fitness data from over 85,000 Australian children aged 9–17 years old showed that, compared with 9-year-old females, 9-year-old males were faster over short sprints (9.8%) and 1 mile (16.6%), could jump 9.5% further from a standing start (a test of explosive power), could complete 33% more push-ups in 30 s and had 13.8% stronger grip [22]. Male advantage of a similar magnitude was detected in a study of Greek children, where, compared with 6-year-old females, 6-year-old males completed 16.6% more shuttle runs in a given time and could jump 9.7% further from a standing position [23]. In terms of aerobic capacity, 6- to 7-year-old males have been shown to have a higher absolute and relative (to body mass) *V*O_2max_ than 6- to 7-year-old females [24]. Nonetheless, while some biological sex differences, probably genetic in origin, are measurable and affect performance pre-puberty, we consider the effect of androgenizing puberty more influential on performance, and have focused our analysis on musculoskeletal differences hereafter.

Secondary sex characteristics that develop during puberty have evolved under sexual selection pressures to improve reproductive fitness and thus generate anatomical divergence beyond the reproductive system, leading to adult body types that are measurably different between sexes. This phenomenon is known as sex dimorphism. During puberty, testes-derived testosterone levels increase 20-fold in males, but remain low in females, resulting in circulating testosterone concentrations at least 15 times higher in males than in females of any age [4, 25]. Testosterone in males induces changes in muscle mass, strength, anthropometric variables and hemoglobin levels [4], as part of the range of sexually dimorphic characteristics observed in humans.

Broadly, males are bigger and stronger than females. It follows that, within competitive sport, males enjoy significant performance advantages over females, predicated on the superior physical capacity developed during puberty in response to testosterone. Thus, the biological effects of elevated pubertal testosterone are primarily responsible for driving the divergence of athletic performances between males and females [4]. It is acknowledged that this divergence has been compounded historically by a lag in the cultural acceptance of, and financial provision for, females in sport that may have had implications for the rate of improvement in athletic performance in females. Yet, since the 1990s, the difference in performance records between males and females has been relatively stable, suggesting that biological differences created by androgenization explain most of the male advantage, and are insurmountable [5, 26, 27].

Table 1 outlines physical attributes that are major parameters underpinning the male performance advantage [28–38]. Males have: larger and denser muscle mass, and stiffer connective tissue, with associated capacity to exert greater muscular force more rapidly and efficiently; reduced fat mass, and different distribution of body fat and lean muscle mass, which increases power to weight ratios and upper to lower limb strength in sports where this may be a crucial determinant of success; longer and larger skeletal structure, which creates advantages in sports where levers influence force application, where longer limb/digit length is favorable, and where height, mass and proportions are directly responsible for performance capacity; superior cardiovascular and respiratory function, with larger blood and heart volumes, higher hemoglobin concentration, greater cross-sectional area of the trachea and lower oxygen cost of respiration [3, 4, 39, 40]. Of course, different sports select for different physiological characteristics—an advantage in one discipline may be neutral or even a disadvantage in another—but examination of a variety of record and performance metrics in any discipline reveals there are few sporting disciplines where males do not possess performance advantage over females as a result of the physiological characteristics affected by testosterone.

**Table 1:** Selected physical difference between untrained/moderately trained males and females. Female levels are set as the reference value

Variable	Magnitude of sex difference (%)	References
Body composition		
Lean body mass	45	Lee et al. [28]
Fat%	− 30	
Muscle mass		
Lower body	33	Janssen et al. [29]
Upper body	40	
Muscle strength		
Grip strength	57	Bohannon et al. [30]
Knee extension peak torque	54	Neder et al. [31]
Anthropometry and bone geometry		
Femur length	9.4	Jantz et al. [32]
Humerus length	12.0	Brinckmann et al. [33]
Radius length	14.6	
Pelvic width relative to pelvis height	− 6.1	
Tendon properties		
Force	83	Lepley et al. [34]
Stiffness	41	
*V*O_2max_		
Absolute values	50	Pate et al. [35]
Relative values	25	
Respiratory function		
Pulmonary ventilation (maximal)	48	Åstrand et al. [36]
Cardiovascular function		
Left ventricular mass	31	Åstrand et al. [36]
Cardiac output (rest)	22	Best et al. [37]
Cardiac output (maximal)	30	Tong et al. [38]
Stroke volume (rest)	43	
Stroke volume (maximal)	34	
Hemoglobin concentration	11	

## Sports Performance Differences Between Males and Females

### An Overview of Elite Adult Athletes

**Fig. 1:**
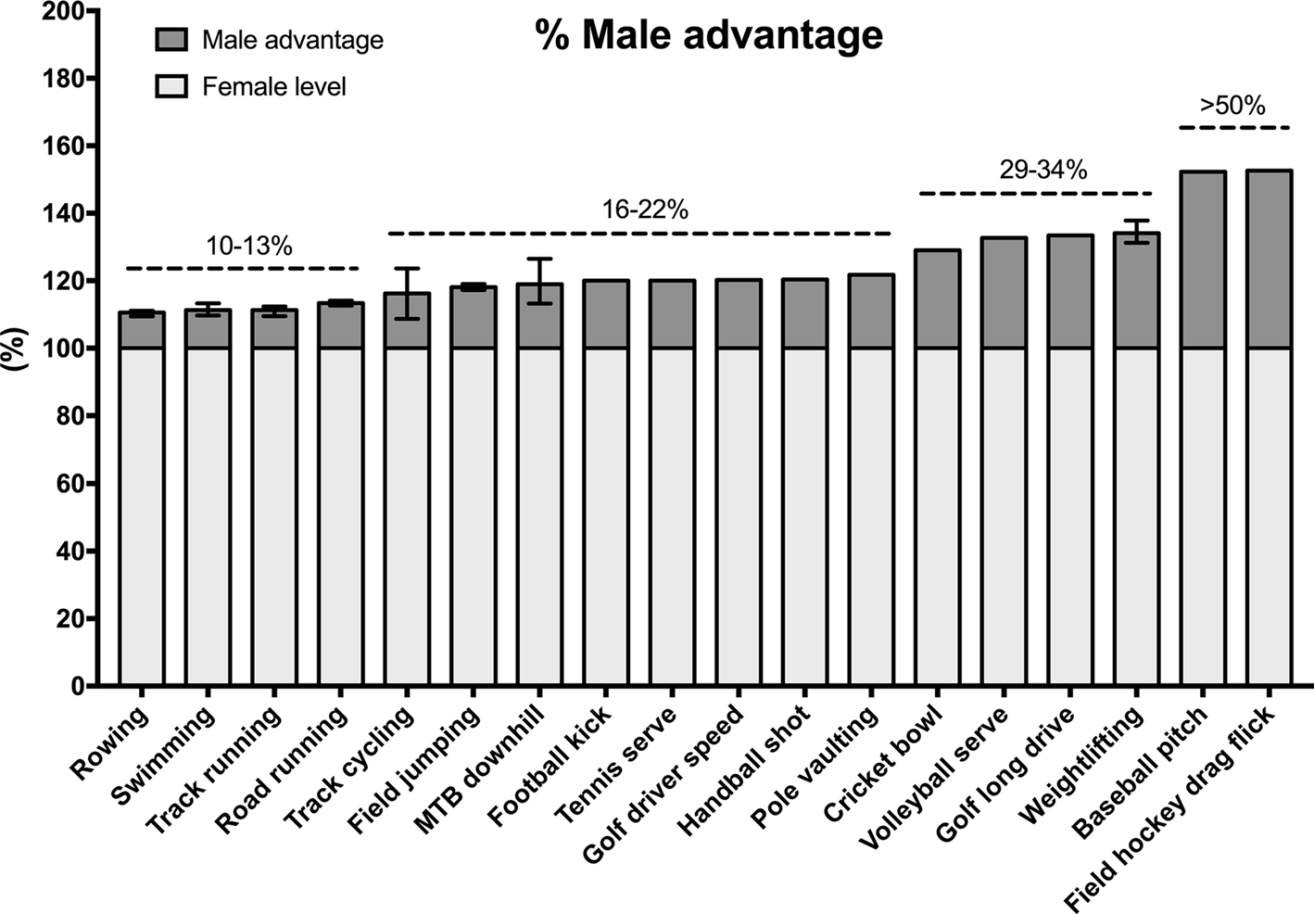
The male performance advantage over females across various selected sporting disciplines. The female level is set to 100%. In sport events with multiple disciplines, the male value has been averaged across disciplines, and the error bars represent the range of the advantage. The metrics were compiled from publicly available sports federation databases and/or tournament/competition records. *MTB* mountain bike

A comparison of adult elite male and female achievements in sporting activities can quantify the extent of the male performance advantage. We searched publicly available sports federation databases and/or tournament/competition records to identify sporting metrics in various events and disciplines, and calculated the performance of males relative to females. Although not an exhaustive list, examples of performance gaps in a range of sports with various durations, physiological performance determinants, skill components and force requirements are shown in Fig. 1.

The smallest performance gaps were seen in rowing, swimming and running (11–13%), with low variation across individual events within each of those categories. The performance gap increases to an average of 16% in track cycling, with higher variation across events (from 9% in the 4000 m team pursuit to 24% in the flying 500 m time trial). The average performance gap is 18% in jumping events (long jump, high jump and triple jump). Performance differences larger than 20% are generally present when considering sports and activities that involve extensive upper body contributions. The gap between fastest recorded tennis serve is 20%, while the gaps between fastest recorded baseball pitches and field hockey drag flicks exceed 50%.

Sports performance relies to some degree on the magnitude, speed and repeatability of force application, and, with respect to the speed of force production (power), vertical jump performance is on average 33% greater in elite men than women, with differences ranging from 27.8% for endurance sports to in excess of 40% for precision and combat sports [41]. Because implement mass differs, direct comparisons are not possible in throwing events in track and field athletics. However, the performance gap is known to be substantial, and throwing represents the widest sex difference in motor performance from an early age [42]. In Olympic javelin throwers, this is manifested in differences in the peak linear velocities of the shoulder, wrist, elbow and hand, all of which are 13–21% higher for male athletes compared with females [43].

The increasing performance gap between males and females as upper body strength becomes more critical for performance is likely explained to a large extent by the observation that males have disproportionately greater strength in their upper compared to lower body, while females show the inverse [44, 45]. This different distribution of strength compounds the general advantage of increased muscle mass in upper body dominant disciplines. Males also have longer arms than females, which allows greater torque production from the arm lever when, for example, throwing a ball, punching or pushing.

### Olympic Weightlifting

In Olympic weightlifting, where weight categories differ between males and females, the performance gap is between 31 and 37% across the range of competitive body weights between 1998 and 2020 (Fig. 1). It is important to note that at all weight categories below the top/open category, performances are produced within weight categories with an upper limit, where strength can be correlated with “fighting weight”, and we focused our analysis of performance gaps in these categories.

To explore strength–mass relationships further, we compared Olympic weightlifting data between equivalent weight categories which, to some extent, limit athlete height, to examine the hypothesis that male performance advantage may be largely (or even wholly) mediated by increased height and lever-derived advantages (Table 2). Between 1998 and 2018, a 69 kg category was common to both males and females, with the male record holder (69 kg, 1.68 m) lifting a combined weight 30.1% heavier than the female record holder (69 kg, 1.64 m). Weight category changes in 2019 removed the common 69 kg category and created a common 55 kg category. The current male record holder (55 kg, 1.52 m) lifts 29.5% heavier than the female record holder (55 kg, 1.52 m). These comparisons demonstrate that males are approximately 30% stronger than females of equivalent stature and mass. However, importantly, male vs. female weightlifting performance gaps increase with increasing bodyweight. For example, in the top/open weight category of Olympic weightlifting, in the absence of weight (and associated height) limits, maximum male lifting strength exceeds female lifting strength by nearly 40%. This is further manifested in powerlifting, where the male record (total of squat, bench press and deadlift) is 65% higher than the female record in the open weight category of the World Open Classic Records. Further analysis of Olympic weightlifting data shows that the 55-kg male record holder is 6.5% stronger than the 69-kg female record holder (294 kg vs 276 kg), and that the 69-kg male record is 3.2% higher than the record held in the female open category by a 108-kg female (359 kg vs 348 kg). This Olympic weightlifting analysis reveals key differences between male and female strength capacity. It shows that, even after adjustment for mass, biological males are significantly stronger (30%) than females, and that females who are 60% heavier than males do not overcome these strength deficits.

**Table 2:** Olympic weightlifting data between equivalent male–female and top/open weight categories

	Sex	Weight (kg)	Height (m)	Combined record (kg)	Strength to weight ratio	Relative performance (%)
2019 record in the 55 kg weight-limited category						
Liao Qiuyun	F	55	1.52	227	4.13	
Om Yun-chol	M	55	1.52	294	5.35	29.5
1998–2018 record in the 69-kg weight-limited category						
Oxsana Slivenko	F	69	1.64	276	4.00	
Liao Hui	M	69	1.68	359	5.20	30.1
Comparative performances for top/open categories (all time heaviest combined lifts)						
Tatiana Kashirina	F	108	1.77	348	3.22	
Lasha Talakhadze	M	168	1.97	484	2.88	39.1

*F* female, *M* male

### Perspectives on Elite Athlete Performance Differences

Figure 1 illustrates the performance gap between adult elite males and adult elite females across various sporting disciplines and activities. The translation of these advantages, assessed as the performance difference between the very best males and very best females, are significant when extended and applied to larger populations. In running events, for example, where the male–female gap is approximately 11%, it follows that many thousands of males are faster than the very best females. For example, approximately 10,000 males have personal best times that are faster than the current Olympic 100 m female champion (World Athletics, personal communication, July 2019). This has also been described elsewhere [46, 47], and illustrates the true effect of an 11% typical difference on population comparisons between males and females. This is further apparent upon examination of selected junior male records, which surpass adult elite female performances by the age of 14–15 years (Table 3), demonstrating superior male athletic performance over elite females within a few years of the onset of puberty.

**Table 3:** Selected junior male records in comparison with adult elite female records

Event	Schoolboy male record	Elite female (adult) record
100 m	10.20 (age 15)	10.49
800 m	1:51.23 (age 14)	1:53.28
1500 m	3:48.37 (age 14)	3:50.07
Long jump	7.85 m (age 15)	7.52 m
Discus throw	77.68 m (age 15)	76.80 m

*M* meters

Time format: minutes:seconds.hundredths of a second

These data overwhelmingly confirm that testosterone-driven puberty, as the driving force of development of male secondary sex characteristics, underpins sporting advantages that are so large no female could reasonably hope to succeed without sex segregation in most sporting competitions. To ensure, in light of these analyses, that female athletes can be included in sporting competitions in a fair and safe manner, most sports have a female category the purpose of which is the protection of both fairness and, in some sports, safety/welfare of athletes who do not benefit from the physiological changes induced by male levels of testosterone from puberty onwards.

### Performance Differences in Non-elite Individuals

The male performance advantages described above in athletic cohorts are similar in magnitude in untrained people. Even when expressed relative to fat-free weight, *V*O_2max_ is 12–15% higher in males than in females [48]. Records of lower-limb muscle strength reveal a consistent 50% difference in peak torque between males and females across the lifespan [31]. Hubal et al. [49] tested 342 women and 243 men for isometric (maximal voluntary contraction) and dynamic strength (one-repetition maximum; 1RM) of the elbow flexor muscles and performed magnetic resonance imaging (MRI) of the biceps brachii to determine cross-sectional area. The males had 57% greater muscle size, 109% greater isometric strength, and 89% greater 1RM strength than age-matched females. This reinforces the finding in athletic cohorts that sex differences in muscle size and strength are more pronounced in the upper body.

Recently, sexual dimorphism in arm force and power was investigated in a punch motion in moderately-trained individuals [50]. The power produced during a punch was 162% greater in males than in females, and the least powerful man produced more power than the most powerful woman. This highlights that sex differences in parameters such as mass, strength and speed may combine to produce even larger sex differences in sport-specific actions, which often are a product of how various physical capacities combine. For example, power production is the product of force and velocity, and momentum is defined as mass multiplied by velocity. The momentum and kinetic energy that can be transferred to another object, such as during a tackle or punch in collision and combat sports are, therefore, dictated by: the mass; force to accelerate that mass, and; resultant velocity attained by that mass. As there is a male advantage for each of these factors, the net result is likely synergistic in a sport-specific action, such as a tackle or a throw, that widely surpasses the sum of individual magnitudes of advantage in isolated fitness variables. Indeed, already at 17 years of age, the average male throws a ball further than 99% of 17-year-old females [51], despite no single variable (arm length, muscle mass etc.) reaching this numerical advantage. Similarly, punch power is 162% greater in men than women even though no single parameter that produces punching actions achieves this magnitude of difference [50].

## Is the Male Performance Advantage Lost when Testosterone is Suppressed in Transgender Women?

The current IOC criteria for inclusion of transgender women in female sports categories require testosterone suppression below 10 nmol/L for 12 months prior to and during competition. Given the IOC’s stated position that the “overriding sporting objective is and remains the guarantee of fair competition” [14]*,* it is reasonable to assume that the rationale for this requirement is that it reduces the male performance advantages described previously to an acceptable degree, thus permitting fair and safe competition. To determine whether this medical intervention is sufficient to remove (or reduce) the male performance advantage, which we described above, we performed a systematic search of the scientific literature addressing anthropometric and muscle characteristics of transgender women. Search terms and filtering of peer-reviewed data are given in Supplementary Table S1.

### Anthropometrics

Given its importance for the general health of the transgender population, there are multiple studies of bone health, and reviews of these data. To summarise, transgender women often have low baseline (pre-intervention) bone mineral density (BMD), attributed to low levels of physical activity, especially weight-bearing exercise, and low vitamin D levels [52, 53]. However, transgender women generally maintain bone mass over the course of at least 24 months of testosterone suppression. There may even be small but significant increases in BMD at the lumbar spine [54, 55]. Some retrieved studies present data pertaining to maintained BMD in transgender women after many years of testosterone suppression. One such study concluded that “BMD is preserved over a median of 12.5 years” [56]. In support, no increase in fracture rates was observed over 12 months of testosterone suppression [54]. Current advice, including that from the International Society for Clinical Densitometry, is that transgender women, in the absence of other risk factors, do not require monitoring of BMD [52, 57]. This is explicable under current standard treatment regimes, given the established positive effect of estrogen, rather than testosterone, on bone turnover in males [58].

Given the maintenance of BMD and the lack of a plausible biological mechanism by which testosterone suppression might affect skeletal measurements such as bone length and hip width, we conclude that height and skeletal parameters remain unaltered in transgender women, and that sporting advantage conferred by skeletal size and bone density would be retained despite testosterone reductions compliant with the IOC’s current guidelines. This is of particular relevance to sports where height, limb length and handspan are key (e.g. basketball, volleyball, handball) and where high movement efficiency is advantageous. Male bone geometry and density may also provide protection against some sport-related injuries—for example, males have a lower incidence of knee injuries, often attributed to low quadriceps (*Q*) angle conferred by a narrow pelvic girdle [59, 60].

### Muscle and Strength Metrics

As discussed earlier, muscle mass and strength are key parameters underpinning male performance advantages. Strength differences range between 30 and 100%, depending upon the cohort studied and the task used to assess strength. Thus, given the important contribution made by strength to performance, we sought studies that have assessed strength and muscle/lean body mass changes in transgender women after testosterone reduction. Studies retrieved in our literature search covered both longitudinal and cross-sectional analyses. Given the superior power of the former study type, we will focus on these.

The pioneer work by Gooren and colleagues, published in part in 1999 [61] and in full in 2004 [62], reported the effects of 1 and 3 years of testosterone suppression and estrogen supplementation in 19 transgender women (age 18–37 years). After the first year of therapy, testosterone levels were reduced to 1 nmol/L, well within typical female reference ranges, and remained low throughout the study course. As determined by MRI, thigh muscle area had decreased by − 9% from baseline measurement. After 3 years, thigh muscle area had decreased by a further − 3% from baseline measurement (total loss of − 12% over 3 years of treatment). However, when compared with the baseline measurement of thigh muscle area in transgender men (who are born female and experience female puberty), transgender women retained significantly higher thigh muscle size. The final thigh muscle area, after three years of testosterone suppression, was 13% larger in transwomen than in the transmen at baseline (*p* < 0.05). The authors concluded that testosterone suppression in transgender women does not reverse muscle size to female levels.

Including Gooren and Bunck [62], 12 longitudinal studies [53, 63–73] have examined the effects of testosterone suppression on lean body mass or muscle size in transgender women. The collective evidence from these studies suggests that 12 months, which is the most commonly examined intervention period, of testosterone suppression to female-typical reference levels results in a modest (approximately − 5%) loss of lean body mass or muscle size (Table 4). No study has reported muscle loss exceeding the − 12% found by Gooren and Bunck after 3 years of therapy. Notably, studies have found very consistent changes in lean body mass (using dual-energy X-ray absorptiometry) after 12 months of treatment, where the change has always been between − 3 and − 5% on average, with slightly greater reductions in the arm compared with the leg region [68]. Thus, given the large baseline differences in muscle mass between males and females (Table 1; approximately 40%), the reduction achieved by 12 months of testosterone suppression can reasonably be assessed as small relative to the initial superior mass. We, therefore, conclude that the muscle mass advantage males possess over females, and the performance implications thereof, are not removed by the currently studied durations (4 months, 1, 2 and 3 years) of testosterone suppression in transgender women. In sports where muscle mass is important for performance, inclusion is therefore only possible if a large imbalance in fairness, and potentially safety in some sports, is to be tolerated.

**Table 4:** Longitudinal studies of muscle and strength changes in adult transgender women undergoing cross-sex hormone therapy

Study	Participants (age)	Therapy	Confirmed serum testosterone levels	Muscle/strength data	Comparison with reference females
Polderman et al. [73]	*N* = 12 TW 18–36 yr (age range)	T suppression + E supplementation	< 2 nmol/L at 4 mo	*LBM* 4 mo − 2.2%	*LBM* 4 mo 16%
Gooren and Bunck [62]	*N* = 19 TW 26 ± 6 yr	T suppression + E supplementation	≤ 1 nmol/L at 1 and 3 yr	*Thigh area* 1 yr − 9% / 3 yr -12%	*Thigh area* 1 yr 16%/3 yr 13%
Haraldsen et al. [63]	*N* = 12 TW 29 ± 8 yr	E supplementation	< 10 nmol/L at 3 mo and 1 yr	*LBM* 3 mo/1 yr—small changes, unclear magnitude	
Mueller et al. [64]	*N* = 84 TW 36 ± 11 yr	T suppression + E supplementation	≤ 1 nmol/L at 1 and 2 yr	*LBM* 1 yr − 4%/2 yr − 7%	
Wierckx et al. [65]	*N* = 53 TW 31 ± 14 yr	T suppression + E supplementation	< 10 nmol/L at 1 yr	*LBM* 1 yr − 5%	*LBM* 1 yr 39%
Van Caenegem et al. [53] (and Van Caenegem et al. [76])	*N* = 49 TW 33 ± 14 yr	T suppression + E supplementation	≤ 1 nmol/L at 1 and 2 yr	*LBM* 1 yr − 4%/2 yr − 0.5% *Grip strength* 1 yr − 7%/2 yr − 9% *Calf area* 1 yr − 2%/2 yr − 4% *Forearm area* 1 yr − 8%/2 yr − 4%	*LBM* 1 yr 24%/2 yr 28% *Grip strength* 1 yr 26%/2 yr 23% *Calf area* 1 yr 16%/2 yr 13% *Forearm area* 1 yr 29%/2 yr 34%
Gava et al. [66]	*N* = 40 TW 31 ± 10 yr	T suppression + E supplementation	< 5 nmol/L at 6 mo and ≤ 1 nmol/L at 1 yr	*LBM* 1 yr − 2%	
Auer et al. [67]	*N* = 45 TW 35 ± 1 (SE) yr	T suppression + E supplementation	< 5 nmol/L at 1 yr	*LBM* 1 yr − 3%	*LBM* 1 yr 27%
Klaver et al. [68]	*N* = 179 TW 29 (range 18–66)	T suppression + E supplementation	≤ 1 nmol/L at 1 yr	*LBM 1 yr* Total − 3% Arm region − 6% Trunk region − 2% Android region 0% Gynoid region − 3% Leg region − 4%	*LBM 1 yr* Total 18% Arm region 28% Leg region 19%
Fighera et al. [69]	*N* = 46 TW 34 ± 10	E supplementation with or without T suppression	< 5 nmol/L at 3 mo ≤ 1 nmol/L at 31 mo	*ALM* 31 mo − 4% from the 3 mo visit	
Scharff et al. [70]	*N* = 249 TW 28 (inter quartile range 23–40)	T suppression + E supplementation	≤ 1 nmol/L at 1 yr	*Grip strength* 1 yr − 4%	*Grip strength* 1 yr 21%
Wiik et al. [71]	*N* = 11 TW 27 ± 4	T suppression + E supplementation	≤ 1 nmol/L at 4 mo and at 1 yr	*Thigh volume* 1 yr − 5% *Quad area* 1 yr − 4% *Knee extension strength* 1 yr 2% *Knee flexion strength* 1 yr 3%	*Thigh volume* 1 yr 33% *Quad area* 26% *Knee extension strength* 41% *Knee flexion strength* 33%

Studies reporting measures of lean mass, muscle volume, muscle area or strength are included. Muscle/strength data are calculated in reference to baseline cohort data and, where reported, reference female (or transgender men before treatment) cohort data. Tack et al. [72] was not included in the table since some of the participants had not completed full puberty at treatment initiation. van Caenegem et al. [76] reports reference female values measured in a separately-published, parallel cohort of transgender men

*N* number of participants, *TW* transgender women, *Yr* year, *Mo* month, *T* testosterone, *E* estrogen. ± Standard deviation (unless otherwise indicated in text), *LBM* lean body mass, *ALM* appendicular lean mass

To provide more detailed information on not only gross body composition but also thigh muscle volume and contractile density, Wiik et al. [71] recently carried out a comprehensive battery of MRI and computed tomography (CT) examinations before and after 12 months of successful testosterone suppression and estrogen supplementation in 11 transgender women. Thigh volume (both anterior and posterior thigh) and quadriceps cross-sectional area decreased − 4 and − 5%, respectively, after the 12-month period, supporting previous results of modest effects of testosterone suppression on muscle mass (see Table 4). The more novel measure of radiological attenuation of the quadriceps muscle, a valid proxy of contractile density [74, 75], showed no significant change in transgender women after 12 months of treatment, whereas the parallel group of transgender men demonstrated a + 6% increase in contractile density with testosterone supplementation.

As indicated earlier (e.g. Table 1), the difference in muscle strength between males and females is often more pronounced than the difference in muscle mass. Unfortunately, few studies have examined the effects of testosterone suppression on muscle strength or other proxies of performance in transgender individuals. The first such study was published online approximately 1 year prior to the release of the current IOC policy. In this study, as well as reporting changes in muscle size, van Caenegem et al. [53] reported that hand-grip strength was reduced from baseline measurements by − 7% and − 9% after 12 and 24 months, respectively, of cross-hormone treatment in transgender women. Comparison with data in a separately-published, parallel cohort of transgender men [76] demonstrated a retained hand-grip strength advantage after 2 years of 23% over female baseline measurements (a calculated average of baseline data obtained from control females and transgender men).

In a recent multicenter study [70], examination of 249 transgender women revealed a decrease of − 4% in grip strength after 12 months of cross-hormone treatment, with no variation between different testosterone level, age or BMI tertiles (all transgender women studied were within female reference ranges for testosterone). Despite this modest reduction in strength, transgender women retained a 17% grip strength advantage over transgender men measured at baseline. The authors noted that handgrip strength in transgender women was in approximately the 25th percentile for males but was over the 90th percentile for females, both before and after hormone treatment. This emphasizes that the strength advantage for males over females is inherently large. In another study exploring handgrip strength, albeit in late puberty adolescents, Tack et al. noted no change in grip strength after hormonal treatment (average duration 11 months) of 21 transgender girls [72].

Although grip strength provides an excellent proxy measurement for general strength in a broad population, specific assessment within different muscle groups is more valuable in a sports-specific framework. Wiik et al., [71] having determined that thigh muscle mass reduces only modestly, and that no significant changes in contractile density occur with 12 months of testosterone suppression, provided, for the first time, data for isokinetic strength measurements of both knee extension and knee flexion. They reported that muscle strength after 12 months of testosterone suppression was comparable to baseline strength. As a result, transgender women remained about 50% stronger than both the group of transgender men at baseline and a reference group of females. The authors suggested that small neural learning effects during repeated testing may explain the apparent lack of small reductions in strength that had been measured in other studies [71].

These longitudinal data comprise a clear pattern of very modest to negligible changes in muscle mass and strength in transgender women suppressing testosterone for at least 12 months. Muscle mass and strength are key physical parameters that constitute a significant, if not majority, portion of the male performance advantage, most notably in those sports where upper body strength, overall strength, and muscle mass are crucial determinants of performance. Thus, our analysis strongly suggests that the reduction in testosterone levels required by many sports federation transgender policies is insufficient to remove or reduce the male advantage, in terms of muscle mass and strength, by any meaningful degree. The relatively consistent finding of a minor (approximately − 5%) muscle loss after the first year of treatment is also in line with studies on androgen-deprivation therapy in males with prostate cancer, where the annual loss of lean body mass has been reported to range between − 2 and − 4% [77].

Although less powerful than longitudinal studies, we identified one major cross-sectional study that measured muscle mass and strength in transgender women. In this study, 23 transgender women and 46 healthy age- and height-matched control males were compared [78]. The transgender women were recruited at least 3 years after sex reassignment surgery, and the mean duration of cross-hormone treatment was 8 years. The results showed that transgender women had 17% less lean mass and 25% lower peak quadriceps muscle strength than the control males [78]. This cross-sectional comparison suggests that prolonged testosterone suppression, well beyond the time period mandated by sports federations substantially reduces muscle mass and strength in transgender women. However, the typical gap in lean mass and strength between males and females at baseline (Table 1) exceeds the reductions reported in this study [78]. The final average lean body mass of the transgender women was 51.2 kg, which puts them in the 90th percentile for women [79]. Similarly, the final grip strength was 41 kg, 25% higher than the female reference value [80]. Collectively, this implies a retained physical advantage even after 8 years of testosterone suppression. Furthermore, given that cohorts of transgender women often have slightly lower baseline measurements of muscle and strength than control males [53], and baseline measurements were unavailable for the transgender women of this cohort, the above calculations using control males reference values may be an overestimate of actual loss of muscle mass and strength, emphasizing both the need for caution when analyzing cross-sectional data in the absence of baseline assessment and the superior power of longitudinal studies quantifying within-subject changes.

### Endurance Performance and Cardiovascular Parameters

No controlled longitudinal study has explored the effects of testosterone suppression on endurance-based performance. Sex differences in endurance performance are generally smaller than for events relying more on muscle mass and explosive strength. Using an age grading model designed to normalize times for masters/veteran categories, Harper [81] analyzed self-selected and self-reported race times for eight transgender women runners of various age categories who had, over an average 7 year period (range 1–29 years), competed in sub-elite middle and long distance races within both the male and female categories. The age-graded scores for these eight runners were the same in both categories, suggesting that cross-hormone treatment reduced running performance by approximately the size of the typical male advantage. However, factors affecting performances in the interim, including training and injury, were uncontrolled for periods of years to decades and there were uncertainties regarding which race times were self-reported vs. which race times were actually reported and verified, and factors such as standardization of race course and weather conditions were unaccounted for. Furthermore, one runner improved substantially post-transition, which was attributed to improved training [81]. This demonstrates that performance decrease after transition is not inevitable if training practices are improved. Unfortunately, no study to date has followed up these preliminary self-reports in a more controlled setting, so it is impossible to make any firm conclusions from this data set alone.

Circulating hemoglobin levels are androgen-dependent [82] and typically reported as 12% higher in males compared with females [4]. Hemoglobin levels appear to decrease by 11–14% with cross-hormone therapy in transgender women [62, 71], and indeed comparably sized reductions have been reported in athletes with DSDs where those athletes are sensitive to and been required to reduce testosterone [47, 83]. Oxygen-carrying capacity in transgender women is most likely reduced with testosterone suppression, with a concomitant performance penalty estimated at 2–5% for the female athletic population [83]. Furthermore, there is a robust relationship between hemoglobin mass and *V*O_2max_ [84, 85] and reduction in hemoglobin is generally associated with reduced aerobic capacity [86, 87]. However, hemoglobin mass is not the only parameter contributing to *V*O_2max_, where central factors such as total blood volume, heart size and contractility, and peripheral factors such as capillary supply and mitochondrial content also plays a role in the final oxygen uptake [88]. Thus, while a reduction in hemoglobin is strongly predicted to impact aerobic capacity and reduce endurance performance in transgender women, it is unlikely to completely close the baseline gap in aerobic capacity between males and females.

The typical increase in body fat noted in transgender women [89, 90] may also be a disadvantage for sporting activities (e.g. running) where body weight (or fat distribution) presents a marginal disadvantage. Whether this body composition change negatively affects performance results in transgender women endurance athletes remains unknown. It is unclear to what extent the expected increase in body fat could be offset by nutritional and exercise countermeasures, as individual variation is likely to be present. For example, in the Wiik et al. study [71], 3 out of the 11 transgender women were completely resistant to the marked increase in total adipose tissue noted at the group level. This inter-individual response to treatment represents yet another challenge for sports governing bodies who most likely, given the many obstacles with case-by-case assessments, will form policies based on average effect sizes.

Altogether, the effects of testosterone suppression on performance markers for endurance athletes remain insufficiently explored. While the negative effect on hemoglobin concentration is well documented, the effects on *V*O_2max_, left ventricular size, stroke volume, blood volume, cardiac output lactate threshold, and exercise economy, all of which are important determinants of endurance performance, remain unknown. However, given the plausible disadvantages with testosterone suppression mentioned in this section, together with the more marginal male advantage in endurance-based sports, the balance between inclusion and fairness is likely closer to equilibrium in weight-bearing endurance-based sports compared with strength-based sports where the male advantage is still substantial.

## Discussion

The data presented here demonstrate that superior anthropometric, muscle mass and strength parameters achieved by males at puberty, and underpinning a considerable portion of the male performance advantage over females, are not removed by the current regimen of testosterone suppression permitting participation of transgender women in female sports categories. Rather, it appears that the male performance advantage remains substantial. Currently, there is no consensus on an acceptable degree of residual advantage held by transgender women that would be tolerable in the female category of sport. There is significant dispute over this issue, especially since the physiological determinants of performance vary across different sporting disciplines. However, given the IOC position that fair competition is the overriding sporting objective [14], any residual advantage carried by transgender women raises obvious concerns about fair and safe competition in the numerous sports where muscle mass, strength and power are key performance determinants.

### Perspectives on Athletic Status of Transgender Women

Whilst available evidence is strong and convincing that strength, skeletal- and muscle-mass derived advantages will largely remain after cross-hormone therapy in transgender women, it is acknowledged that the findings presented here are from healthy adults with regular or even low physical activity levels [91], and not highly trained athletes. Thus, further research is required in athletic transgender populations.

However, despite the current absence of empirical evidence in athletic transgender women, it is possible to evaluate potential outcomes in athletic transgender women compared with untrained cohorts. The first possibility is that athletic transgender women will experience similar reductions (approximately − 5%) in muscle mass and strength as untrained transgender women, and will thus retain significant advantages over a comparison group of females. As a result of higher baseline characteristics in these variables, the retained advantage may indeed be even larger. A second possibility is that by virtue of greater muscle mass and strength at baseline, pre-trained transgender women will experience larger relative decreases in muscle mass and strength if they converge with untrained transgender women, particularly if training is halted during transition. Finally, training before and during the period of testosterone suppression may attenuate the anticipated reductions, such that relative decreases in muscle mass and strength will be smaller or non-existent in transgender women who undergo training, compared to untrained (and non-training) controls.

It is well established that resistance training counteracts substantial muscle loss during atrophy conditions that are far more severe than testosterone suppression. For example, resistance exercise every third day during 90-days bed rest was sufficient to completely offset the 20% reduction in knee extensor muscle size noted in the resting control subjects [92]. More relevant to the question of transgender women, however, is to examine training effects in studies where testosterone has been suppressed in biological males. Kvorning et al. investigated, in a randomized placebo-controlled trial, how suppression of endogenous testosterone for 12 weeks influenced muscle hypertrophy and strength gains during a training program (3 days/week) that took place during the last 8 weeks of the 3-month suppression period [93]. Despite testosterone suppression to female levels of 2 nmol/L, there was a significant + 4% increase in leg lean mass and a + 2% increase in total lean body mass, and a measurable though insignificant increase in isometric knee extension strength. Moreover, in select exercises used during the training program, 10RM leg press and bench press increased + 32% and + 17%, respectively. While some of the training adaptations were lower than in the placebo group, this study demonstrates that training during a period of testosterone suppression not only counteracts muscle loss, but can actually increase muscle mass and strength.

Males with prostate cancer undergoing androgen deprivation therapy provide a second avenue to examine training effects during testosterone suppression. Testosterone levels are typically reduced to castrate levels, and the loss of lean mass has typically ranged between − 2 and − 4% per year [77], consistent with the findings described previously in transgender women. A recent meta-analysis concluded that exercise interventions including resistance exercise were generally effective for maintaining muscle mass and increasing muscle strength in prostate cancer patients undergoing androgen deprivation therapy [94]. It is important to emphasize that the efficacy of the different training programs may vary. For example, a 12-week training study of prostate cancer patients undergoing androgen deprivation therapy included drop-sets to combine heavy loads and high volume while eliciting near-maximal efforts in each set [95]. This strategy resulted in significantly increased lean body mass (+ 3%), thigh muscle volume (+ 6%), knee extensor 1RM strength (+ 28%) and leg press muscle endurance (+ 110%).

In addition to the described effects of training during testosterone suppression, the effect of training prior to testosterone suppression may also contribute to the attenuation of any muscle mass and strength losses, via a molecular mechanism referred to as ‘muscle memory’ [96]. Specifically, it has been suggested that myonuclei acquired by skeletal muscle cells during training are maintained during subsequent atrophy conditions [97]. Even though this model of muscle memory has been challenged recently [98], it may facilitate an improved training response upon retraining [99]. Mechanistically, the negative effects of testosterone suppression on muscle mass are likely related to reduced levels of resting protein synthesis [100], which, together with protein breakdown, determines the net protein balance of skeletal muscle. However, testosterone may not be required to elicit a robust muscle protein synthesis response to resistance exercise [100]. Indeed, relative increases in muscle mass in men and women from resistance training are comparable, despite marked differences in testosterone levels [101], and the acute rise in testosterone apparent during resistance exercise does not predict muscle hypertrophy nor strength gains [102]. This suggests that even though testosterone is important for muscle mass, especially during puberty, the maintenance of muscle mass through resistance training is not crucially dependent on circulating testosterone levels.

Thus, in well-controlled studies in biological males who train while undergoing testosterone reduction, training is protective of, and may even enhance, muscle mass and strength attributes. Considering transgender women athletes who train during testosterone suppression, it is plausible to conclude that any losses will be similar to or even smaller in magnitude than documented in the longitudinal studies described in this review. Furthermore, pre-trained transgender women are likely to have greater muscle mass at baseline than untrained transgender women; it is possible that even with the same, rather than smaller, relative decreases in muscle mass and strength, the magnitude of retained advantage will be greater. In contrast, if pre-trained transgender women undergo testosterone suppression while refraining from intense training, it appears likely that muscle mass and strength will be lost at either the same or greater rate than untrained individuals, although there is no rationale to expect a weaker endpoint state. The degree of change in athletic transgender women is influenced by the athlete’s baseline resistance-training status, the efficacy of the implemented program and other factors such as genetic make-up and nutritional habits, but we argue that it is implausible that athletic transgender women would achieve final muscle mass and strength metrics that are on par with reference females at comparable athletic level.

### The Focus on Muscle Mass and Strength

We acknowledge that changes in muscle mass are not always correlated in magnitude to changes in strength measurements because muscle mass (or total mass) is not the only contributor to strength [103]. Indeed, the importance of the nervous system, e.g. muscle agonist activation (recruitment and firing frequency) and antagonist co-activation, for muscle strength must be acknowledged [104]. In addition, factors such as fiber types, biomechanical levers, pennation angle, fascicle length and tendon/extracellular matrix composition may all influence the ability to develop muscular force [105]. While there is currently limited to no information on how these factors are influenced by testosterone suppression, the impact seems to be minute, given the modest changes noted in muscle strength during cross-hormone treatment.

It is possible that estrogen replacement may affect the sensitivity of muscle to anabolic signaling and have a protective effect on muscle mass [106] explaining, in part, the modest change in muscle mass with testosterone suppression and accompanying cross-hormone treatment. Indeed, this is supported by research conducted on estrogen replacement therapy in other targeted populations [107, 108] and in several different animal models, including mice after gonadectomy [109] and ovariectomy [110].

In terms of other performance proxies relevant to sports performance, there is no research evaluating the effects of transgender hormone treatment on factors such as agility, jumping or sprint performance, competition strength performance (e.g. bench press), or discipline-specific performance. Other factors that may impact sports performance, known to be affected by testosterone and some of them measurably different between males and females, include visuospatial abilities, aggressiveness, coordination and flexibility.

### Testosterone-Based Criteria for Inclusion of Transgender Women in Female Sports

The appropriate testosterone limit for participation of transgender women in the female category has been a matter of debate recently, where sports federations such as World Athletics recently lowered the eligibility criterion of free circulating testosterone (measured by means of liquid chromatography coupled with mass spectrometry) to < 5 nmol/L. This was based, at least in part, on a thorough review by Handelsman et al. [4], where the authors concluded that, given the nonoverlapping distribution of circulating testosterone between males and females, and making an allowance for females with mild hyperandrogenism (e.g. with polycystic ovary syndrome), the appropriate testosterone limit should be 5 rather than 10 nmol/L.

From the longitudinal muscle mass/strength studies summarised here, however, it is apparent that most therapeutic interventions result in almost complete suppression of testosterone levels, certainly well below 5 nmol/L (Table 4). Thus, with regard to transgender women athletes, we question whether current circulating testosterone level cut-off can be a meaningful decisive factor, when in fact not even suppression down to around 1 nmol/L removes the anthropometric and muscle mass/strength advantage in any significant way.

In terms of duration of testosterone suppression, it may be argued that although 12 months of treatment is not sufficient to remove the male advantage, perhaps extending the time frame of suppression would generate greater parity with female metrics. However, based on the studies reviewed here, evidence is lacking that this would diminish the male advantage to a tolerable degree. On the contrary, it appears that the net loss of lean mass and grip strength is not substantially decreased at year 2 or 3 of cross-hormone treatment (Table 4), nor evident in cohorts after an average 8 years after transition. This indicates that a plateau or a new steady state is reached within the first or second year of treatment, a phenomenon also noted in transgender men, where the increase in muscle mass seems to stabilise between the first and the second year of testosterone treatment [111].

## Conclusions

We have shown that under testosterone suppression regimes typically used in clinical settings, and which comfortably exceed the requirements of sports federations for inclusion of transgender women in female sports categories by reducing testosterone levels to well below the upper tolerated limit, evidence for loss of the male performance advantage, established by testosterone at puberty and translating in elite athletes to a 10–50% performance advantage, is lacking. Rather, the data show that strength, lean body mass, muscle size and bone density are only trivially affected. The reductions observed in muscle mass, size, and strength are very small compared to the baseline differences between males and females in these variables, and thus, there are major performance and safety implications in sports where these attributes are competitively significant. These data significantly undermine the delivery of fairness and safety presumed by the criteria set out in transgender inclusion policies, particularly given the stated prioritization of fairness as an overriding objective (for the IOC). If those policies are intended to preserve fairness, inclusion and the safety of biologically female athletes, sporting organizations may need to reassess their policies regarding inclusion of transgender women.

From a medical-ethical point of view, it may be questioned as to whether a requirement to lower testosterone below a certain level to ensure sporting participation can be justified at all. If the advantage persists to a large degree, as evidence suggests, then a stated objective of targeting a certain testosterone level to be eligible will not achieve its objective and may drive medical practice that an individual may not want or require, without achieving its intended benefit.

The research conducted so far has studied untrained transgender women. Thus, while this research is important to understand the isolated effects of testosterone suppression, it is still uncertain how transgender women athletes, perhaps undergoing advanced training regimens to counteract the muscle loss during the therapy, would respond. It is also important to recognize that performance in most sports may be influenced by factors outside muscle mass and strength, and the balance between inclusion, safety and fairness therefore differs between sports. While there is certainly a need for more focused research on this topic, including more comprehensive performance tests in transgender women athletes and studies on training capacity of transgender women undergoing hormone therapy, it is still important to recognize that the biological factors underpinning athletic performance are unequivocally established. It is, therefore, possible to make strong inferences and discuss potential performance implications despite the lack of direct sport-specific studies in athletes. Finally, since athlete safety could arguably be described as the immediate priority above considerations of fairness and inclusion, proper risk assessment should be conducted within respective sports that continue to include transgender women in the female category.

If transgender women are restricted within or excluded from the female category of sport, the important question is whether or not this exclusion (or conditional exclusion) is necessary and proportionate to the goal of ensuring fair, safe and meaningful competition. Regardless of what the future will bring in terms of revised transgender policies, it is clear that different sports differ vastly in terms of physiological determinants of success, which may create safety considerations and may alter the importance of retained performance advantages. Thus, we argue against universal guidelines for transgender athletes in sport and instead propose that each individual sports federation evaluate their own conditions for inclusivity, fairness and safety.

### Supplementary Information

Below is the link to the electronic supplementary material.

Supplementary file1 (PDF 99 KB)
