# Supplementary material for: Transgender Women in the Female Category of Sport: Perspectives on Testosterone Suppression and Performance Advantage
Source: Sports Med. 2020 Dec 8;51(2):199–214. doi: 10.1007/s40279-020-01389-3 (PMC7846503; doi:10.1007/s40279-020-01389-3)
Supplement: Supplementary file 1 — Supplementary file1 (PDF 99 KB) [file 40279_2020_1389_MOESM1_ESM.pdf]

Supplementary Table S1 Literature review search details (PubMed accessed 08.03.2020)

Article title: Transgender women in the female category of sport: Perspectives on testosterone suppression and performance advantage

Journal: Sports Medicine

Authors: Emma N. Hilton [1] and Tommy R. Lundberg [2, 3]

Affiliations:

1) Faculty of Biology, Medicine and Health, University of Manchester, Manchester, UK.
2) Department of Laboratory Medicine, Division of Clinical Physiology, Karolinska Institutet, Stockholm, Sweden.
3) Unit of Clinical Physiology, Karolinska University Hospital, Stockholm, Sweden.

Correspondence:
Tommy R. Lundberg, PhD
E-mail: **tommy.lundberg@ki.se**

Search terms

Search term 1	[AND]	Search term 2
trans [AND] sex [OR] gender [OR] gender identity [OR] (gender [AND] identity)<br>transgender [OR] transgender persons [OR](transgender [AND] persons)<br>transwoman [OR] (trans [AND] woman)<br>transwomen [OR] (trans [AND] women)<br>transsexual [OR] transsexuals		muscle [OR] muscles [OR] strength<br>bone [OR] bones [OR] height<br>body composition [OR] (body [AND] composition)<br>sport [OR] sports [OR] performance<br>exercise [OR] physical activity [OR] (physical [AND] activity)<br>athlete [OR] athletes

A full list of references is available on request.

Sorting strategy

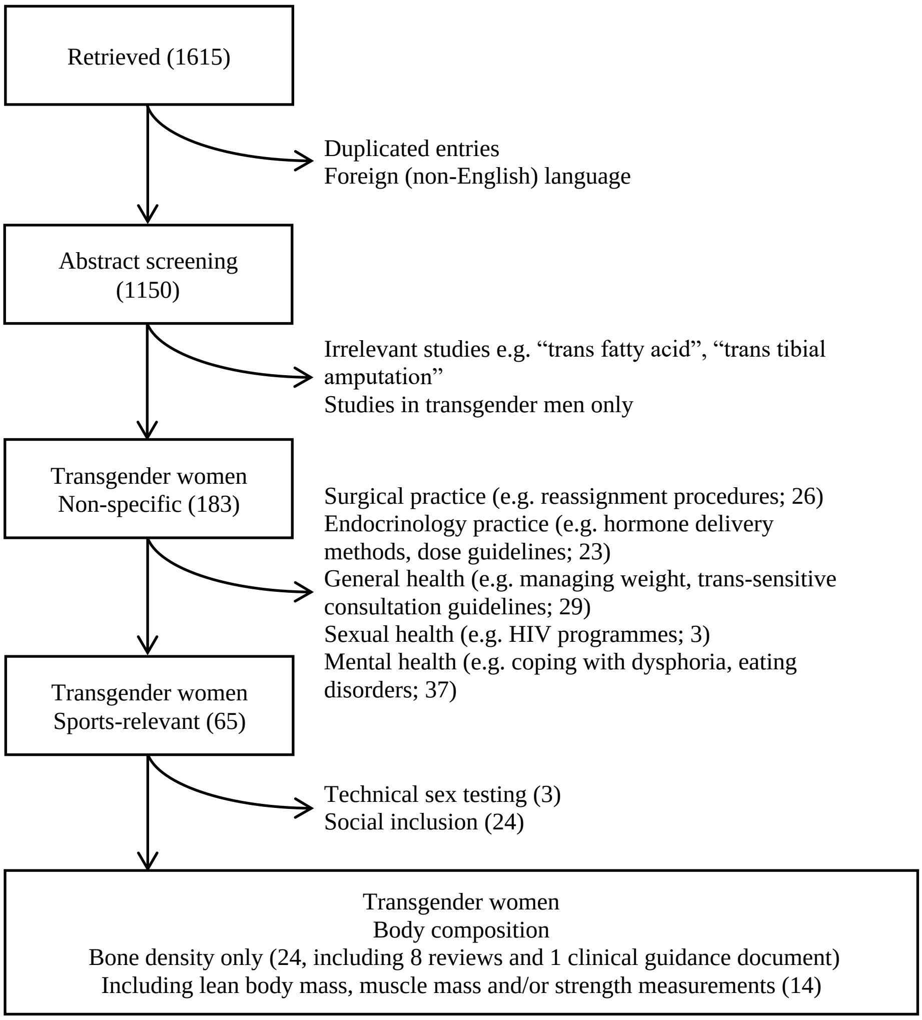

Declarations

Funding: None

Conflicts of interest: Emma N Hilton and Tommy R Lundberg declare that they have no conflict of interest.

Authorship contributions: Both authors (ENH and TRL) were involved in the conception and design of this paper, and both authors drafted, revised and approved the final version of the paper.

Ethics approval: Not applicable

Informed consent: Not applicable

Data availability: Available upon request
